# Comparison of the Mineral and Nutraceutical Profiles of Elephant Garlic (*Allium ampeloprasum* L.) Grown in Organic and Conventional Fields of Valdichiana, a Traditional Cultivation Area of Tuscany, Italy

**DOI:** 10.3390/biology10101058

**Published:** 2021-10-18

**Authors:** Stefano Loppi, Riccardo Fedeli, Giulia Canali, Massimo Guarnieri, Stefano Biagiotti, Andrea Vannini

**Affiliations:** 1 Department of Life Sciences, University of Siena, 53100 Siena, Italy; riccardo.fedeli@student.unisi.it (R.F.); giulia.canali@student.unisi.it (G.C.); massimo.guarnieri@unisi.it (M.G.); andrea.vannini@unisi.it (A.V.); 2 BAT Center—Interuniversity Center for Studies on Bioinspired Agro-Environmental Technology, University of Naples ‘Federico II’, 80138 Naples, Italy; 3 Pegaso Online University, 53045 Montepulciano, Italy; stefano.biagiotti@unipegaso.it

**Keywords:** great-headed garlic, antioxidants, polyphenols, soluble sugars, elemental content

## Abstract

**Simple Summary:**

In Valdichiana, an agricultural area of Tuscany (C Italy), an antique landrace of elephant garlic (*A. ampeloprasum* L.) locally known as “Aglione della Valdichiana” has been cultivated for a long time, and has been recently recognized as a traditional agri-food product of Tuscany and of Italy. Two methods of cultivation of elephant garlic are currently in use in Valdichiana: conventional and organic, the latter not making use of mineral fertilizers and chemical pesticides, even if the cultivation of elephant garlic is a low impact one. This paper aimed at testing if there are differences in the mineral and nutraceutical profiles of elephant garlic cultivated conventionally and organically in the Valdichiana area. Our results indicated only small differences and no evidence of healthier food or superior nutraceutical quality for organically grown elephant garlic.

**Abstract:**

In the Valdichiana area (Tuscany, Italy) an ancient native landrace of elephant garlic (*Allium ampeloprasum* L.), locally known as “Aglione della Valdichiana”, has long been cultivated. The aim of this study was to check whether there are differences in the mineral and nutraceutical profiles of the Aglione della Valdichiana cultivated conventionally and organically. Based on the analysis by ICP-MS of a wide array of major, minor, essential, and non-essential trace elements as well as rare earth elements, and the evaluation of the content of polyphenols, flavonoids, antioxidants, soluble proteins, soluble sugars, and starch, as well as the weight and water content, it was concluded that differences in the mineral and nutraceutical profiles of organically and conventionally grown bulbs were very limited. Only a statistically (*p* < 0.05) higher concentration of Cd (+2620%), Co (+113%), Mn (+55%), Rb (+180%), and Sb (+180%), as well as glucose (+37%) in conventionally cultivated bulbs emerged. Cadmium was the only element slightly higher than in the “reference plant,” but with a negligible risk (three orders of magnitude lower) for human health based on consumption. It is concluded that we failed to find evidence of healthier food or a higher nutraceutical quality for organically cultivated elephant garlic.

## 1. Introduction

*Allium ampeloprasum* L. is a species widely distributed in the Mediterranean area and is traditionally considered to be represented by four different groups: leek, kurrat, pearl onion, and elephant garlic [1]. However, a molecular study [2] has suggested that leek, kurrat, and pearl onion (tetraploids) constitute a distinct taxon, for which the restoration of the name *A. porrum* L. was proposed, while *A. ampeloprasum* L. (hexaploid) should refer only to elephant garlic.

*A. ampeloprasum* is much bigger (about three times) than common garlic (*Allium sativum* L.), and hence the name elephant, giant, or great-headed garlic [3]. A key feature of *A. ampeloprasum* is a reduced content of fibers and sulphur-containing compounds (alliin-related compounds) compared to common garlic [4], which makes this species preferred over garlic in cooking, having a similar but more delicate taste, and a higher digestibility. For this reason, *A. ampeloprasum* is also known as “kissing garlic” or “garlic for people who do not like garlic” [5]. Overall, elephant garlic is a leek-like plant, but producing cloves that are bigger and have a milder flavor than those of common garlic.

The phytochemical profile of *A. ampeloprasum* has been the subject of several studies, mostly aimed at testing its biological activity [5,6,7], but the mineral and nutraceutical profiles of elephant garlic are still poorly known.

In Valdichiana, an agricultural area of Tuscany (C Italy), an antique landrace of elephant garlic locally known as “Aglione della Valdichiana” has been cultivated for a long time and has been recently recognized as a traditional agri-food product of Tuscany and of Italy. Two methods of cultivation of elephant garlic are currently in use in Valdichiana: conventional and organic, the latter not making use of mineral fertilizers and chemical pesticides, even if the cultivation of elephant garlic is a low impact one.

Currently, there is a pressing move towards organic farming to limit the massive use of external inputs of fertilizers and pesticides (see, e.g., the Farm to Fork strategy of the EU), and organic agriculture seeks to address this challenge by using practices that are considered more environment friendly and nature-based alternatives [8,9]. Although there is evidence that organic farming contributes positively to the agro-biodiversity and the natural biodiversity [10], the alleged fact that organic products are healthier and have a higher nutritional value remains unclear and matter of debate [11,12]. Nevertheless, there is growing interest in investigating landraces and ecotypes, since these can have important quality traits, as they were selected before the massive use of external inputs [13].

This paper aims at testing if there are differences in the mineral and nutraceutical profiles of elephant garlic cultivated conventionally and organically in the Valdichiana area. The data relative to 12 out of 37 mineral elements have already been presented in Vannini et al. [14].

## 2. Materials and Methods

### 2.1. Plant Material

Four cultivation fields of elephant garlic were selected in Valdichiana thanks to the collaboration of the local association for the protection and promotion of elephant garlic from Valdichiana, which selected two farms that cultivate the elephant garlic in a conventional way and two in a certified organic way (fully organically cultivated fields for >5 years). The agronomic practices carried out at the conventional fields involve the use of inorganic (19%P_2_O_5_, 29%SO_3_; 250 kg/ha) and organic (15%N, 5%P_2_O_5_, 5%K_2_O, 26%SO_3_, 10%C; 167 kg/ha) chemical fertilizers, chemical weeding (38 g/L Pendimethalin; 10 L/ha), and mechanical hoeing, while at the organic fields there is manual hoeing, manual weeding, manuring (2000 kg/ha) and allowed chemical organic fertilizers (4%N, 8%P_2_O_5_, 10%K_2_O, 8%SO_3_, 20%C; 500 kg/ha). The four fields are less than 15 km apart and have fairly similar and homogeneous pedo-climatic conditions. Field size varies between 1000 m^2^ and 9000 m^2^. Each of the four farmers provided 50 randomly selected bulbs from the growing season 2019. Samples of elephant garlic were peeled, and either freeze-dried and pulverized with mortar and pestle for the elemental analysis or finely chopped and ground in a mortar for the nutraceutical analysis. Five homogeneous samples (sampling units) were obtained for each field (experimental unit).

### 2.2. Elemental Analysis

The samples (250 mg of dry weight material) were mineralized with a mixture of 3 mL of 70% HNO_3_ and 1 mL of 30% H_2_O_2_ in a microwave digestion system (Milestone Ethos 900) at 280 °C and 55 bar. The content of major, minor, essential, and non-essential trace elements, as well as rare earth elements (REEs), was determined by inductively coupled plasma mass spectrometry (Perkin Elmer NexION 350 spectrometer; Waltham, MA, USA) and expressed on a dry weight basis (either mg/kg or μg/kg dw). The analytical quality was checked with the certified Standard Reference Material GBW 07604 “Leaves of Poplar” and showed recoveries in the range 94–106%. The analytical precision was >95% for all elements. For each sample, five technical replicates were measured.

### 2.3. Nutraceutical Analysis

#### 2.3.1. Soluble Proteins

Ground samples of ca. 100 mg were homogenized in 4 mL of deionized water and centrifuged at 4000 rpm for 5 min.; the supernatant (0.2 mL) was then added to 0.8 mL of Bradford solution (Sigma-Aldrich, St. Louis, MO, USA). Samples were read at 595 nm with a UV-Vis spectrophotometer (Agilent 8453; Santa Clara, CA, USA). Quantification was achieved using a calibration curve (10–100 µg/mL) with bovine albumin (Sigma-Aldrich), and the results were expressed as mg of bovine albumin equivalent on a fresh weight basis (mg BAE/g fw).

#### 2.3.2. Polyphenols

Polyphenols were measured according to the method proposed by Henríquez et al. [15], with minor modifications. Ground samples of ca. 100 mg were homogenized in 4 mL of 70% acetone and centrifuged at 4000 rpm for 5 min. The extract (0.5 mL) was then added to 0.125 mL of Folin-Denis’ reagent (Sigma-Aldrich), 0.750 mL of a saturated NaCO_3_ solution, and 0.950 mL of deionized water. The resulting solution was then left at 36° for 30 min and then centrifuged again. Samples were read at 750 nm with a UV-Vis spectrophotometer (Agilent 8453). Quantification was done with a calibration curve (30-300 µg/mL) of gallic acid (Sigma-Aldrich), and the results were expressed as mg of gallic acid equivalent on a fresh weight basis (mg GAE/g fw).

#### 2.3.3. Flavonoids

Flavonoids were measured according to the method proposed by Heimler et al. [16]. Ground samples of ca. 500 mg were homogenized in 2 mL of 80% ethanol and then centrifuged at 15,000 rpm for 5 min. The supernatant (500 µL) was added to 45 µL of a 5% NaNO_2_ solution and 300 µL of deionized water. Then, 45 µL of a 10% AlCl_3_ solution, 300 µL of a 1M NaOH solution, and 300 µL of deionized water were added. Samples were read at 510 nm with a UV-Vis spectrophotometer (Agilent 8453). Quantification was done with a calibration curve (5–200 µg/mL) of quercetin (Sigma-Aldrich), and the results were expressed as mg of quercetin equivalent on a fresh weight basis (mg QE/g fw). 

#### 2.3.4. Antioxidant Power

Grounded samples of ca. 100 mg were homogenized in 2 mL of 80% ethanol and then centrifuged at 15,000 rpm for 5 min. The supernatant (100 µL) was then added to 1 mL of a DPPH solution prepared by dissolving 3.9 mg of this compound in 100 mL of methanol/water (80:20 v/v). Samples were then left in the dark for 1 h and their absorbance read at 517 nm with a UV-Vis spectrophotometer (Agilent 8453). A blank was prepared by dissolving 100 µL of a 80% ethanol solution in 1 mL of a 80% methanol solution. Results were expressed as % antiradical activity (ARA%) according to the formula: ARA %= 100 × (1 − (sample/control)), where control indicates the absorbance of the reagents only.

#### 2.3.5. Starch

Starch was measured according to the method proposed by Clément et al. [17], with minor modifications. Ground samples of ca. 100 mg were homogenized in 4 mL of dimethylsulfoxide (DMSO) and then 0.5 mL of 8 M HCl was added. The mixture was left at 60 °C for 30 min, and, after cooling, 0.5 mL of 8 M NaOH was added. At the end, the solution was brought to a final volume of 10 mL with deionized water. The sample was centrifuged at 4000 rpm for 5 min and 0.5 mL of the supernatant were added to 2.5 mL of a Lugol solution (HCl 0.05 M—0.03% I_2_, 0.06% KI) and allowed to react for 15 min. Absorbance was read at 605 nm with a UV-Vis spectrophotometer (Agilent 8453). The starch content was quantified using a calibration curve (10.0–440 μg/mL) prepared with pure starch (Merck). The results were expressed on a fresh weight basis (mg/g fw).

#### 2.3.6. Soluble Sugars and Total Sweetness Index (TSI)

Ground samples of ca. 100 mg were homogenized in 2 mL of deionized water and then centrifuged at 15,000 rpm for 5 min. The supernatant was filtered at 0.45 µm using a syringe filter and then directly analyzed by HPLC (Waters 600 system) equipped with a Waters 2410 refractive index detector. Sugars separation was granted using deionized water as mobile phase, eluted at 0.5 mL/min, and a Waters Sugar-Pak I ion-exchange column (6.5 × 300 mm) kept at 90 °C using an external temperature controller (Waters Column Heater Module). The quantification of sucrose, glucose, and fructose was achieved by means of calibration curves prepared by dissolving the three pure sugars (Merck) in deionized water at concentrations of 0.1–20 mg/mL. Results were expressed on a fresh weight basis (mg/g fw). A total sweetness index (TSI) was calculated according to the formula proposed by Magwaza and Opara [18].

### 2.4. Statistical Analysis

To disentangle the effect of cultivation on the mineral and nutraceutical profile, a linear mixed-effect model (LMEM) was fitted for each variable, with cultivation method as fixed effect and cultivation field as random effect. LMEMs are an extension of simple linear models to allow both fixed and random effects and are particularly useful when the data have a hierarchical structure [19]. For model validation, scatterplots of the residual and fitted values were used to check for homoscedasticity, and normal probability (qqnorm) plots as well as the Shapiro–Wilk test to check for normality. Models were fitted using the restricted maximum likelihood (REML) estimation, and the significance of the models was checked with type III ANOVA using the Satterthwaite method [20]. In the case of some values below the detection limit (Cd, Sb), an isometric log-ratio expectation-maximization (ilr-EM) algorithm was used to obtain meaningful data for the statistical analysis [21]. Since for these two latter elements LMEM residuals did not match a normal distribution, a generalized linear mixed-effect model (GLMM) was run using the Gamma distribution. All statistical computations were run using the free software R [22].

## 3. Results and Discussion

Notwithstanding a very wide array of major, minor, essential, and non-essential trace elements as well as rare earth elements was determined in organically and traditionally grown samples of elephant garlic cultivated in Valdichiana (Table 1), only a few elements showed a statistically significant difference between the two groups, namely Cd, Co, Mn, Rb, and Sb, which were all higher in the conventional growing fields. All these elements can be found in fertilizers, especially phosphate ones [23,24,25,26]. Additionally, there is also evidence that the addition of fertilizers can increase the mobility of certain elements in the soil and hence enhance plant uptake [27,28,29].

Comparing the content of Cd, Co, Mn, Rb, and Sb in bulbs of elephant garlic with those reported for the reference plant [30], it emerged that concentrations of Co, Mn, Rb, and Sb were within the typical values (0.2, 200, 50, and 0.1 mg/kg dw, respectively), while the concentration of Cd was higher than the 0.05 mg/kg dw reported for the reference plant, but only in the bulbs cultivated conventionally. However, a deeper inspection of Cd concentrations showed that only one cultivation field had high Cd values, while the other was very close to the values of the reference plant (0.21 ± 0.03 mg/kg dw vs. 0.06 ± 0.01 mg/kg dw). Slightly high Cd values in bulbs of elephant garlic from the Valdichiana area were also reported by Vannini et al. [14], who showed, however, that at these concentrations the health risk from dietary consumption of this vegetable is negligible (three orders of magnitude lower than a dose that would pose a risk).

A comparison of major and minor essential element concentrations with those from elephant garlic cultivated in other countries such as Spain [31] and India [32,33] showed that *A. ampeloprasum* from the Valdichiana area is rich in important elements such as P, K, Mg, Fe, and Zn (Table 2), which are fundamental for dietary intake [34].

The fresh weight and the water content of elephant garlic cloves were measured based on the so-called “market conditions”, i.e., fresh weight after 1–2 months from the harvest, implying a weight reduction of ca. 5–10% and a water loss of ca. 20%. These two parameters did not differ among organically and conventionally grown bulbs (Table 3), with an overall mean fresh weight of 16.8 g and a mean water content of 61%.

The content of soluble proteins, polyphenols, and flavonoids, along with the antioxidant power, did not differ between organic and conventional bulbs (Table 3), with mean values in agreement with those reported for *A. ampeloprasum* grown worldwide, e.g., S Italy [5], Spain [31], and California [7].

The analysis of starch and soluble sugars, as well as the total sweetness index, did not show differences between elephant garlic grown organically and conventionally, except for a statistically (*p* < 0.05) higher content of glucose in conventional fields (Table 3). The fact that the total sweetness index does not differ significantly (*p* > 0.05) between organically grown and conventional elephant garlic allows us to deduce that the lower glucose content does not have a negative impact on the quality of the organic elephant garlic. Additionally, despite some variability in the content of the single sugars (sucrose, glucose, fructose), the mean total sugar content was consistent with that reported by Ulianych et al. [35] for *A. ampeloprasum* grown in Ukraine.

Compared with several landraces of common garlic (*Allium sativum* L.) from Italy [36], cloves of elephant garlic grown in Valdichiana showed a ca. 10-fold higher weight, a higher content of glucose and fructose, and a lower content of sucrose. Furthermore, comparison with common garlic from China [37] confirmed the higher fructose and the lower sucrose content of our elephant garlic; moreover, a similar content of soluble proteins and flavonoids also emerged, contrasting with the lower starch and polyphenol content in elephant garlic. The lower content of starch, sucrose, and glucose, and the higher content of fructose in elephant garlic from Valdichiana emerged also when compared with common garlic cultivated in Azerbaijan [38].

The production of organic food is strongly driven by market-based considerations [39], and organic food, accounting for 6% of the food sold in the USA in 2020, is the fastest growing sector of the food industry, with a remarkable 12.8% increase in 2020, totaling ca. 56.4 billion USD (Organic Trade Association, www.ota.com accessed on 20 August 2021). This notable increase is likely due to the common belief of consumers that organic food is healthier than conventionally grown foods. Additionally, there is also the environmental motivation, determined by the assumption that organic farming is beneficial for biodiversity. However, notwithstanding the need to reduce the use of fertilizers and pesticides of synthetic origin, these issues are quite controversial, and studies are far from being conclusive. Some LCA-based studies have shown the environmental benefits of organic food consumption, while others have demonstrated the need for a much higher amount of arable land to achieve a similar yield [40,41,42]. In the EU, ca. 8.5 % of the total utilized arable land is cultivated organically [43], and the European Commission, in the framework of the European Green Deal, has launched the “Farm to Fork” strategy, which foresees that 25% of the total agricultural land will be cultivated organically by 2030 [44].

It is quite obvious that food production and consumption may have a great impact on the environment and human health, but the question whether organic food is really healthier and of higher quality is still matter of debate. Organic food is often associated with a lower content of pesticide residues and potentially toxic elements compared with conventional food [45,46,47], and the results of our study on elephant garlic from Valdichiana confirm this point, having shown a higher content of some elements in conventionally grown bulbs, even if only Cd emerged as slightly enriched, and at levels absolutely negligible for health risk for consumers based on a daily intake. Organic food is sometimes reported to have a higher antioxidant content, likely caused by increased attack by pathogens, which may stimulate the production of elevated levels of plant secondary metabolites such as polyphenolics and flavonoids [48], but the scientific community is still undecided whether organic food is more nutritious than conventionally grown food, and there is a large body of evidence that the nutraceutical value of organic food is not different from conventional food [48,49,50]. Our results are consistent with this latter view, since we did not observe any difference in the content of polyphenols, flavonoids, antioxidants, soluble proteins, starch, sucrose, or fructose, as well as total sweetness index, between organic and conventional *A. ampeloprasum* bulbs. Additionally, the weight and water content of cloves, which are important parameters for the producers and the resellers, were non-dissimilar between organic and conventional fields.

## 4. Conclusions

There is a growing interest in and attention on organic farming, where the use of mineral fertilizers and chemical pesticides is not allowed, and for the resulting organic food, which is generally regarded as healthier and of better quality than conventional food by the consumers, who are willing to pay more for such products. However, clear scientific evidence of these advantages is very often missing, as in the case of our study, which focused on the mineral and nutraceutical profiles of elephant garlic, a low impact plant, cultivated conventionally and organically in the Valdichiana area of Tuscany. Our results, despite the wide array of elements investigated, only showed slightly higher concentrations of a few elements, namely Cd, Co, Mn, Rb, and Sb, in bulbs cultivated conventionally, but only Cd was higher than the content reported for a theoretical “reference plant,” and pose a negligible risk for human health based on consumption. Moreover, differences did not emerge for other nutraceutical parameters, such as polyphenols, flavonoids, antioxidants, soluble proteins, starch, sucrose, and fructose, as well as the total sweetness index, between bulbs grown organically and conventionally.

## Figures and Tables

**Table 1:** Mean (M) concentration and standard error (SE) (mg/kg dw) of major, minor, trace, and rare-earth elements measured in elephant garlic bulbs cultivated organically and conventionally in Valdichiana. * = statistically significant (*p* < 0.05) difference.

	Organic		Conventional	
	M	SE	M	SE
Major essential elements				
K	13627	663	13100	591
S	9691	691	11008	378
P	2970	171	2780	128
Mg	905	16	972	34
Ca	585	48	548	40
Minor and trace essential elements				
Na	68	6	97	18
Fe	50	2	52	2
Zn	24.4	2.9	23.5	2.4
Mn *	7.5	0.3	11.6	0.5
Cu	4.0	0.4	4.6	0.4
Ni	3.4	0.9	3.9	0.5
V	0.08	0.01	0.06	0.01
Co *	0.024	0.002	0.051	0.004
Trace non-essential elements				
Sr	10.0	0.8	8.1	0.3
Ba	1.8	0.2	1.6	0.1
Rb *	1.5	0.2	4.2	0.5
Pb	0.26	0.01	0.28	0.02
Cr	0.13	0.04	0.21	0.04
U	0.070	0.003	0.074	0.003
Cd *	0.005	0.001	0.136	0.028
Sb *	0.005	0.001	0.014	0.005
As	<0.001	-----	<0.001	-----
Tl	<0.001	-----	<0.001	-----
Rare-earth elements				
Ce	0.033	0.004	0.026	0.002
La	0.012	0.001	0.013	0.002
Nd	0.007	0.001	0.007	0.001
Pr	0.002	0.001	0.002	0.001
Sm	0.002	0.001	0.002	0.001
Eu	<0.001	-----	<0.001	-----
Gd	<0.001	-----	<0.001	-----
Tb	<0.001	-----	<0.001	-----
Dy	<0.001	-----	<0.001	-----
Ho	<0.001	-----	<0.001	-----
Er	<0.001	-----	<0.001	-----
Tm	<0.001	-----	<0.001	-----
Yb	<0.001	-----	<0.001	-----
Lu	<0.001	-----	<0.001	-----

**Table 2:** Range of major and minor element concentrations (mg/kg dw) in elephant garlic cultivated in Valdichiana (present study), Spain [31], and India [32,33].

	**Valdichiana**	**Spain**	**India**
Major essential elements			
P	2270–015	------------	430–1184
K	10635–17194	1466–5332	3100–4576
Mg	834–1153	89–164	100–186
Ca	321–833	302–817	125–630
Minor essential elements			
Fe	41.4–57.9	2.0–9.2	11.0–12.1
Na	15–203	436–671	67–90
Zn	12.9–44.2	0.3–16.7	4.0–6.5
Cu	2.4–6.6	0.5–2.2	1.0–9.6

**Table 3:** Nutraceutical parameters measured in elephant garlic bulbs cultivated organically and conventionally in Valdichiana. All values not in % are in mg/g fw, except the fresh weight of the cloves which is in grams. TSI = total sweetness index. * = statistically significant (*p* < 0.05) difference.

	**Organic**		**Conventional**	
	**M**	**SE**	**M**	**SE**
Fresh weight	19.9	8.5	13.6	4.6
Water %	61.4	0.1	61.1	0.1
Soluble proteins	8.4	2.8	10.2	0.2
Polyphenols	2.3	0.3	3.0	0.3
Flavonoids	1.5	0.2	2.0	0.2
ARA%	16.7	2.2	18.1	0.3
Starch	3.9	0.1	3.8	0.1
Sucrose	9.1	3.8	6.5	0.2
Glucose *	5.9	0.2	8.1	0.3
Fructose	13.3	0.2	15.3	0.1
TSI	33.6	5.5	35.7	0.2

## Data Availability

The raw data presented in this study are available on request from the corresponding author. The data are not yet publicly available since the project is still ongoing.
